# High frequency of *KRAS* and *EGFR* mutation profiles in *BRAF*-negative thyroid carcinomas in Indonesia

**DOI:** 10.1186/s13104-022-06260-4

**Published:** 2022-12-12

**Authors:** Didik Setyo Heriyanto, Vincent Laiman, Nikko Vanda Limantara, Widyan Putra Anantawikrama, Fara Silvia Yuliani, Rita Cempaka, Sumadi Lukman Anwar

**Affiliations:** 1 Department of Anatomical Pathology, Faculty of Medicine, Public Health, and Nursing, Universitas Gadjah Mada-Dr. Sardjito Hospital, Farmako Street, Yogyakarta, 55281 Indonesia; 2 Department of Pharmacology and Therapy, Faculty of Medicine, Public Health, and Nursing, Universitas Gadjah Mada, Yogyakarta, 55281 Indonesia; 3 Department of Surgery, Subdivision of Oncology Surgery; Faculty of Medicine, Public Health, and Nursing, Universitas Gadjah Mada-Dr. Sardjito Hospital, Yogyakarta, 55281 Indonesia

**Keywords:** BRAF, EGFR, Indonesia, KRAS, Thyroid cancer

## Abstract

**Objective:**

Thyroid cancer incidence has steadily increased in Indonesia. However, data on Kirsten rat sarcoma virus (*KRAS*) and *EGFR* mutations in thyroid cancer in Indonesia remain unavailable, except for *BRAF-V600E*, the most common *BRAF* gene mutation. This study aimed to analyze *KRAS* and *EGFR* mutation profiles in *BRAF-V600E* negative thyroid cancer samples.

**Results:**

*BRAF-V600E* mutations were found in papillary thyroid carcinomas in 40.3% patients with mean age of 53 years old. In *BRAF-V600E*-negative samples, 41.3% had *KRAS* mutations with mean age of 55.5 years old. KRAS mutation was found in 52.6% of follicular carcinomas and 47.4% of papillary thyroid carcinomas. Additionally, 45.7% had *EGFR* mutations in patients with mean age of 50.5 years old. EGFR mutation was found in 71.4% of papillary thyroid carcinoma and 28.6% of follicular carcinoma. Nearly half of the *BRAF-V6*00E negative thyroid carcinoma samples harbored either *KRAS* or *EGFR* mutations. This finding suggests that in *BRAF-V600E* negative thyroid carcinoma samples, testing for *RAS* and *EGFR* mutation may be warranted for further therapeutic consideration.

**Supplementary Information:**

The online version contains supplementary material available at 10.1186/s13104-022-06260-4.

## Introduction

Thyroid cancer is the most common endocrine cancer in the world, with Indonesia ranked 9th position in 2010 [1, 2]. The currently available prognostic factors in thyroid cancer are the TNM classification of malignant tumors (TNM) staging, age, gender, and histopathology profile (including capsular invasion and angioinvasion) [3]. Although clinical assessment of these factors are known, the frequency and behavior of thyroid cancer vary greatly, ranging from frequent and generally asymptomatic to larger and more advanced papillary tumors.

Mutation in B-Raf proto-oncogene (*BRAF*) is the gene most often disrupted in thyroid cancer [4]. *BRAF-V600E* mutation increases disease progression risk in thyroid cancer and correlates with poor prognosis [5, 6]. Following *BRAF* mutations, Ras proteins are related to follicular-patterned neoplasms and are the second most common genetic alteration seen in thyroid carcinomas [7]. The RAS proteins are encoded by three human RAS genes: Kirsten rat sarcoma viral (*KRAS*), neuroblastoma (*NRAS*) and Harvey (*HRAS*). However, despite extensive research, their profile remains largely uncharacterized especially in Indonesia [8]. Epidermal growth factor receptor (*EGFR*), on the other hand, have been implicated in various cancer pathogenesis, including thyroid cancer [9, 10]. *EGFR* was also implied to be important for thyroid carcinoma proliferation and metastasis, and high *EGFR* level was reported in malignant thyroid cancer with wild type *BRAF* [10–12]. This suggest that *EGFR* mutation profile in thyroid cancer should not be overlooked. Overall, investigation for different mutation profiles on thyroid carcinomas is still required to understand the key mutation that occurred.

With high mutation prevalence in thyroid cancer, a preoperative approach is required to assist clinicians in determining the extent to which surgical intervention and adjuvant therapy are required. However, research focusing on mutation profile, including the presence of *KRAS* and *EGFR* mutations in addition to *BRAF* mutations in thyroid cancer, is still rarely described, particularly in Indonesia. This study is expected to be a reference in making diagnostic and treatment decisions in thyroid cancer.

## Main text

### Materials and methods

#### Study design

This study was an retrospective observational study design. Samples were obtained from the archive between 2013 and 2019 and used to investigate BRAF, KRAS, and EGFR mutations in thyroid carcinoma samples. Paraffin blocks from patients histopathologically diagnosed with primary thyroid carcinoma (papillary thyroid carcinoma of all variants, and follicular thyroid carcinoma) were included. Samples were obtained from Department of Anatomical Pathology, Faculty of Medicine, Public Health, and Nursing, Universitas Gadjah Mada, and CITO Pathology Laboratory. Histological evaluation was reviewed by two pathologists and noninvasive follicular thyroid neoplasm with papillary-like nuclear features (NIFTP) samples were excluded. The paraffin blocks were required to contain more than 50% area of tumor. Exclusion criteria were damaged paraffin blocks and paraffin blocks stored at temperatures other than room temperature. This study initially collected 87 primary thyroid carcinoma cases. Of those, highly degraded DNA samples were excluded, leaving only 77 paraffin blocks meeting the criteria. The samples were then examined for *BRAF*, *KRAS* and *EGFR* mutation at Department of Anatomical Pathology; Faculty of Medicine, Public Health, and Nursing, Universitas Gadjah Mada. All specimens were collected under approval of ethical committee at Medical and Health Research Ethics Committee (MHREC) Faculty of Medicine, Public Health, and Nursing Universitas Gadjah Mada-Dr. Sardjito General Hospital (Ref. No.: KE/FK/0411/EC/2020).

#### DNA extraction

DNA extraction was performed from paraffin blocks using tissue genomic DNA mini kit (GeneAll^®^ Clinic SV mini Cat. 108-101, Seoul, Korea) according to the manufacturer’s instructions.

#### Real-time polymerase chain reaction (qRT-PCR)

DNA samples were assayed using AmoyDx *BRAF-V600* mutation detection kit (Amoy Diagnostics, Xiamen, China) [13]; AmoyDx human *EGFR* gene mutations fluorescence PCR diagnostic kit (Amoy Diagnostics, Xiamen, China), detecting T970M, L858R, L861Q, S7681, G719S, G719A, G719C, 3 insertions in exon 20, and 19 deletions in exon 19 [14]; and AmoyDx *KRAS* Mutations Detection Kit (Amoy Diagnostics, Xiamen, China) [15]. Quantitative PCR was performed using Bioneer Exicycler™ 96 Real-Time Quantitative Thermal Block. The assays and PCR condition were as manufacturer’s recommendation. Briefly, region of interest within the DNA sequence is amplified using the polymerase chain reaction, in which the DNA of the mutant gene is amplified by specific primers and detected by novel probes. The amplified region of interest is heated gradually until the double stranded DNA breaks. The cycle threshold (Ct) values at which the signal is detected above the fluorescence background of the respective mutation testing kit were used to determine whether a sample is positive or negative. Internal control reagents are intended to detect the presence of inhibitors, which can result in false-negative results. The real-time PCR was performed with 1 cycle denaturation at 95 °C for 5 min, 15 cycles annealing at 95 °C for 25 s, 64 °C for 20 s, and 72 °C for 20 s, and 31 cycles extension at 93 °C for 25 s, 60 °C for 35 s, and 72 °C for 20 s.

#### Data analysis

All data presented were made using Microsoft Excel 2020 for Mac OSX. Categorical data were expressed as frequency and percentage, and quantitative data were expressed as mean.

## Results

### Clinicopathologic characteristics of the thyroid carcinoma samples

Characteristics of the samples are shown in Table 1. A total of 37 (48.1%) samples were from males and 40 (51.9%) samples were from females. The mean age of the patients was 53.6 ± 13.4 years old. The histopathological types were 57 samples of papillary type (74%) and 20 samples of follicular type (26%). The papillary type was consisted of 32 classic variant (56.1%), 19 follicular variant (33.3%), and three samples of tall cell and columnar cells variant (5.3% and 5.3%, respectively).

**Table 1:** Characteristics of the study samples

Characteristics	N (%)
Gender	
Male	37 (48.1)
Female	40 (51.9)
Age, years	
Mean ± standard deviation	53.6 ± 13.4
Thyroid carcinoma variants	
Papillary thyroid carcinoma	57 (74.0)
Classic variant	32 (56.1)
Follicular variant	19 (33.3)
Tall cell variant	3 (5.3)
Columnar cell variant	3 (5.3)
Follicular carcinoma	20 (26)

#### BRAF-V600E mutation status in thyroid carcinoma samples

A total of 31 (40.3%) samples showed *BRAF* positive mutation consisted of 5 males and 26 females (Table 2). The mean age of the patients was 53.0 ± 14.7 years old. Histopathological types of *BRAF* positive mutation samples were of papillary type, with classic variant of 26 samples (76.5%) and follicular of 5 (14.7%) samples.

**Table 2:** Characteristics and *BRAF-V600E* mutation status in thyroid carcinoma samples

Characteristics	*BRAF-V600E*	
	+, n (%)	−, n (%)
Frequency	31 (40.3)	46 (59.7)
Male	5 (16.1)	32 (69.6)
Female	26 (83.9)	14 (30.4)
Mean age, years ± standard deviation	53 ± 14.7	54.0 ± 12.7
Thyroid carcinoma variants		
Papillary thyroid carcinoma	31	26 (56.5)
Classic variant	26 (76.5)	6 (23.1)
Follicular variant	5 (14.7)	14 (53.9)
Tall cell variant	0	3 (11.5)
Columnar cell variant	0	3 (11.5)
Follicular carcinoma	0	20 (43.5)
Capsular invasion+	18 (58.1)	38 (82.6)
Capsular invasion−	13 (41.9)	8 (17.4)
Vascular invasion+	10 (32.3)	32 (69.6)
Vascular invasion−	21 (67.7)	14 (30.4)

#### KRAS and EGFR mutation were detected in BRAF-V600E negative samples

Further examination of the *BRAF*-negative samples revealed that 19 (41.3%) were *KRAS* positive, with 15 of males and 4 of females (Table 3). The mean age was 55.5 ± 12.4 years old. Histopathological types of *KRAS* positive mutation samples were of papillary type 9 (47.4%) samples and follicular type 10 (52.6%) samples. The papillary type consisted of two (10.5%) classic variant samples, three (15.8%) follicular samples, three (15.8%) tall cell samples, and one (5.2%) columnar cell variant. Capsular invasion was observed in 84.2% of *KRAS* positive samples, while vascular invasion was observed in 78.9% of samples.

**Table 3:** Characteristics and mutation status of *KRAS* and *EGFR* in *BRAF-V600E* negative thyroid carcinoma samples

Characteristics	*KRAS*		*EGFR*	
	+, n (%)	−, n (%)	+, n (%)	−, n (%)
Frequency	19 (41.3)	27 (58.7)	21 (45.7)	25 (54.3)
Male	15 (78.9)	17 (63.0)	13 (61.9)	27 (45.8)
Female	4 (21.1)	10 (37.0)	8 (38.1)	32 (54.2)
Mean age, years ± standard deviation	55.5 ± 12.4	51.9 ± 12.9	50.5 ± 12.5	55.7 ± 12.6
Thyroid carcinoma variants				
Papillary thyroid carcinoma	9 (47.4)	17 (63.0)	15 (71.4)	11 (44)
Classic variant	2 (10.5)	4 (14.8)	4 (19.0)	2 (8.0)
Follicular variant	3 (15.8)	11 (40.7)	9 (42.9)	5 (20.0)
Tall cell variant	3 (15.8)	0	0	3 (12.0)
Columnar cell variant	1 (5.3)	2 (7.4)	2 (9.5)	1 (5.0)
Follicular carcinoma	10 (52.6)	10 (37.0)	6 (28.6)	14 (56)
Anaplastic carcinoma	0	0	0	0
Capsular invasion+	16 (84.2)	22 (81.5)	17 (81.0)	21 (85.0)
Capsular invasion−	3 (15.8)	5 (18.5)	4 (19.0)	4 (15.0)
Vascular invasion+	15 (78.9)	17 (63.0)	12 (57.1)	20 (80.0)
Vascular invasion−	4 (21.1)	10 (37.0)	9 (42.9)	5 (20.0)

*BRAF* negative samples also showed *EGFR* positive mutation in 21 (45.7%) samples consisted of 13 males and 8 female samples. The mean age was 50.5 ± 12.5 years old. Histopathological types of *EGFR* positive samples were 15 papillary type (71.4%), and six follicular type (28.6%). The papillary type consisted of four classic variant samples (19%), nine follicular (42.9%) samples, and two (9.5%) columnar cell variants. Capsular invasion was found in 81.0% of *EGFR* positive samples, with vascular invasion in 57.1% of the samples. Notably we also identified one papillary thyroid carcinoma of follicular variant with both *KRAS* and *EGFR* mutation (Additional file 1: Table S2).

## Discussion

The main findings of this study were (1) The thyroid cancers samples were consisted of papillary type carcinoma (74.0%) and follicular type carcinoma (26.0%), (2) *BRAF-V600E* mutation were found in 40.3% of thyroid carcinoma samples, (3) *KRAS* mutation was found in 41.3% of *BRAF* negative thyroid carcinoma samples, with predominant follicular type, and (4) *EGFR* mutation was observed in 45.7% of *BRAF* negative thyroid carcinoma samples, with predominant papillary type.

We observed that thyroid cancers samples were mainly of papillary type. Papillary thyroid carcinoma was the most common carcinoma arising from the thyroid gland [16]. In South Korea, for example, papillary thyroid carcinoma accounted for the greatest age-standardized incidence rate [16]. Even though it is considered as malignancy with good prognosis, recent study with 15-year median follow up showed significantly lower survival percentage in papillary thyroid carcinoma with *BRAF* mutation [17]. Therefore, determining the thyroid carcinoma mutation profile is important in determining patient prognosis.

We observed that *BRAF* mutation was found in 40.3% of all thyroid samples, which is comparable to previous studies in Southeast Asia. Previous study in Philippine reported 38.46% of *BRAF* mutation identified in thyroid samples [18]. Another study in Hanoi showed that 56.3% of all thyroid samples have *BRAF* mutation [19]. These findings, along with the prognostic value of *BRAF* mutation, may have contributed the high thyroid cancer deaths (3103 deaths) in Indonesia, despite that papillary thyroid cancer had good survival rate [20]. The number of deaths came at third position following China and India. *BRAF* mutations were associated with thyroid cancer pathogenesis, leading to unregulated cell growth and carcinogenesis as well as extrathyroidal extension and advanced stage [18, 21]. This implies that identifying mutations in thyroid cancer is critical for better diagnosis and, potentially, better treatment.

The RAS proteins transmit extracellular signals that promote cell proliferation, differentiation, and survival [22, 23]. The *EGFR* signaling plays a role in signaling the downstream *BRAF*. Therefore, we conduct testing on *KRAS* and *EGFR* mutation in thyroid cancer patients who did not harbor *BRAF* mutation. We observed that *KRAS* mutation occurred in 41.3% of the *BRAF* negative thyroid cancer samples, and 52.6% of it was found in follicular thyroid carcinoma. Similar finding was reported in Japan in which 57% of follicular carcinomas harbored RAS mutations [8]. Another study in Saudi Arabia assessing proliferative thyroid lesions reported that 32% of mutations affected the KRAS codon 61 [24]. Consistently, we reported high KRAS prevalence and this may be higher since we reported KRAS mutations only in BRAF-negative thyroid cancer samples. Point mutations in the RAS or BRAF proto-oncogenes were detected in almost 70% of papillary thyroid carcinomas in nearly mutually exclusive manner [25]. This further shows that BRAF-negative samples are prone to have other mutations, including RAS mutations. In addition, this could be the factor contributing to poor prognosis in follicular thyroid carcinoma, as *RAS* mutation is linked to significant lower outcome [26]. Therefore, establishing the necessary mutation testing steps in thyroid carcinoma is important to determine the need for early intervention.

We also observed that 45.7% of *BRAF* negative thyroid carcinoma samples had *EGFR* mutation, with 71.4% was found in papillary thyroid carcinoma. This finding is similar to a study in United States with most of *EGFR* mutation was found in papillary thyroid carcinoma (46.2%), followed by follicular type (29.6%) [27]. *EGFR* is important for extracellular signal transduction from the surface into cells to mediate cell proliferation and apoptosis [27]. Its overexpression is common in breast, lung, and bladder cancer. Fisher et al. found that *EGFR* mutations correlates with advanced stage and lymph node metastases which may contribute to papillary thyroid carcinoma aggressiveness [27]. These suggest that *EGFR* mutation profile in thyroid carcinoma should not be excluded.

Interestingly, coexistence mutations (both *KRAS* and *EGFR* mutations) were found in 1 of our *BRAF* negative thyroid carcinoma sample. In non-small cell lung cancer (NSCLC), for example, coexistence mutations of *KRAS* and *EGFR* are relatively rare [28]. This phenomenon was suggested to occur due to heterogeneity in malignant cells where some subpopulations of the cells had different mutations. The *EGFR* coexistence with *KRAS* mutation resulted in significantly shorter progression free survival [29]. Although *KRAS* and *EGFR* mutations have been well investigated in NSCLC, however, study of these mutations on thyroid carcinoma is still limited. These findings emphasize the importance of identifying *KRAS* and *EGFR* mutations following the most common genetic alteration in thyroid cancer, *BRAF*.

Our study highlights the frequent *BRAF*, *KRAS*, and *EGFR* mutations in thyroid carcinomas. Of those *BRAF-V600E* negative samples, almost half had either *KRAS* or *EGFR* mutations. Although differentiated thyroid cancers have favourable prognosis, about 30% will experience recurrent disease after 10 years [30]. *BRAF, KRAS, and EGFR* mutations in recurrent thyroid cancer may benefit from targeted therapies and further study on these mutations with their risk of recurrence, distant metastasis, and overall prognosis in thyroid cancers may be required.

## Conclusion

We discovered that either *KRAS* or *EGFR* mutations can be found in nearly half of the *BRAF* negative thyroid carcinoma samples. Thyroid carcinoma-related death is still a burden in Indonesia. These findings suggest that additional mutational pathways, such as *KRAS* and *EGFR*, should be tested in *BRAF*-*V600E* negative thyroid carcinoma samples. The breakthrough in molecular testing guidelines might provide help in determining the prognosis of thyroid cancer and might aid clinicians in determining early intervention for better outcome.

## Limitation

Due to sample availability, this study only comprised a small number of specimens. In addition, the data obtained in this study were pathology data, whereas with clinical and survival data, a better association can be concluded. Future research should have a large enough sample size to establish enough statistical power for analysis.

## Supplementary Information

**Additional file 1: Table S1.** List of study samples. **Table S2.** *KRAS* and *EGFR* mutation status in *BRAF-V600E* negative samples.

## Data Availability

All data generated or analysed during this study are included in this published article.

## Abbreviations

BRAF: B-Raf proto-oncogene serine/threonine kinase
EGFR: Epidermal growth factor receptor
KRAS: Kirsten rat sarcoma virus
NSCLC: Non-small cell lung cancer
qRT-PCR: Real-time polymerase chain reaction
TKI: Tyrosine kinase inhibitor
TNM: TNM classification of malignant tumors
