# Supplementary material for: High frequency of KRAS and EGFR mutation profiles in BRAF-negative thyroid carcinomas in Indonesia
Source: BMC Res Notes. 2022 Dec 12;15:369. doi: 10.1186/s13104-022-06260-4 (PMC9743704; doi:10.1186/s13104-022-06260-4)
Supplement: Supplementary file 1 — Additional file 1: Table S1. List of study samples. Table S2. KRAS and EGFR mutation status in BRAF-V600E negative samples. [file 13104_2022_6260_MOESM1_ESM.docx]

**Additional Information**

**High Frequency of *KRAS* and *EGFR* Mutation Profiles in *BRAF*-Negative Thyroid Carcinomas in Indonesia**

Didik Setyo Heriyanto^1*^, Vincent Laiman^1^, Nikko Vanda Limantara^1^, Widyan Putra Anantawikrama^1^, Fara Silvia Yuliani^2^, Rita Cempaka^1^, Sumadi Lukman Anwar^3^

^1^Department of Anatomical Pathology; Faculty of Medicine, Public Health, and Nursing; Universitas Gadjah Mada – Dr. Sardjito Hospital/55281, Yogyakarta, Indonesia

^2^Department of Pharmacology and Therapy; Faculty of Medicine, Public Health, and Nursing; Universitas Gadjah Mada/55281, Yogyakarta, Indonesia

^3^Department of Surgery, Subdivision of Oncology Surgery; Faculty of Medicine, Public Health, and Nursing; Universitas Gadjah Mada – Dr. Sardjito Hospital/55281, Yogyakarta, Indonesia

*** Corresponding Author**

*Didik Setyo Heriyanto, MD, PhD*

Department of Anatomical Pathology, Faculty of Medicine, Public Health, and Nursing, Universitas Gadjah Mada, Farmako Street, Yogyakarta 55281, Indonesia.

Telephone: +62 274 540460. E-mail: didik_setyoheriyanto@mail.ugm.ac.id

Table S1. List of study samples

| **ID SAMPLE** | **Gender** | **Age (y)** | **Pathological Diagnosis** | **Capsular invasion** | **Vascular invasion** | **Tumor size (greatest dimension, cm)** | **BRAF** | **KRAS** | **EGFR** | **Remarks** |
| --- | --- | --- | --- | --- | --- | --- | --- | --- | --- | --- |
| T1 | Male | 54 | PTC Follicular | + | + | 4 | - | + | Exon 19 deletion |  |
| T2 | Female | 36 | PTC Classic | - | - | 7 | + |  |  |  |
| T3 | Female | 56 | PTC Classic | + | - | 3 | + |  |  |  |
| T6 | Male | 72 | Follicular carcinoma | + | + | 8 | - | + |  |  |
| T7 | Female | 35 | PTC Classic | - | - | 2 | + |  |  |  |
| T8 | Female | 43 | PTC Classic | - | - | 4 | + |  |  |  |
| T11 | Female | 52 | PTC Classic | + | + | 5 | + |  |  |  |
| T12 | Male | 64 | PTC Follicular | + | - | 12 | + |  |  |  |
| T17 | Male | 50 | Follicular carcinoma | + | + | 10 | - |  | Exon 19 deletion |  |
| T19 | Female | 39 | PTC Classic | - | - | 3 | + |  |  |  |
| T20 | Male | 65 | PTC Follicular | + | - | 13 | - | + |  |  |
| T21 | Female | 29 | PTC Classic | - | - | 3 | - |  | Exon 19 deletion |  |
| T27 | Male | 73 | Follicular carcinoma | + | + | 5 | - | + |  |  |
| T28 | Female | 62 | PTC Classic | + | + | 4 | + |  |  |  |
| T31 | Male | 52 | Follicular carcinoma | + | + | 3 |  |  |  | Necrosis |
| T32 | Female | 51 | PTC Classic | + | - | 6 |  |  |  | Low DNA |
| T33 | Female | 69 | PTC Classic | + | - | 4 |  |  |  | Broken paraffin block |
| T35 | Female | 43 | PTC Follicular | + | - | 6 | - |  | Exon 19 deletion |  |
| T39 | Male | 65 | PTC Follicular | + | - | 3 | + |  |  |  |
| T40 | Female | 33 | PTC Classic | - | - | 7 | - | + |  |  |
| T48 | Female | 32 | PTC Classic | - | - | 2 | + |  |  |  |
| T49 | Male | 59 | Follicular carcinoma | + | + | 14 | - | + |  |  |
| T52 | Female | 48 | PTC Classic | - | - | 4 | + |  |  |  |
| T54 | Female | 80 | PTC Follicular | + | - | 2 |  |  |  | Necrosis |
| T58 | Male | 46 | PTC Classic | - | - | 2 | - |  | 18 |  |
| T59 | Female | 25 | PTC Classic | - | - | 3 | - | + |  |  |
| T60 | Female | 64 | PTC Classic | + | - | 6 | + |  |  |  |
| T62 | Female | 43 | PTC Classic | + | + | 5 | + |  |  |  |
| T63 | Male | 53 | PTC Tall cell | + | + | 8 | - | + |  |  |
| T64 | Male | 67 | Follicular carcinoma | + | + | 12 | - |  |  |  |
| T66 | Female | 63 | PTC Classic | + | - | 3 | + |  |  |  |
| T67 | Male | 58 | PTC Follicular | - | - | 4 |  |  |  | Necrosis |
| T69 | Male | 71 | PTC Follicular | + | - | 9 | - |  | Exon 19 deletion |  |
| T70 | Male | 56 | Follicular carcinoma | + | + | 3 |  |  |  | Low DNA |
| T71 | Female | 49 | PTC Follicular | + | - | 4 |  |  |  | Low DNA |
| T72 | Female | 62 | Follicular carcinoma | + | + | 11 | - | + |  |  |
| T73 | Female | 39 | PTC Follicular | - | - | 6 | - |  | 21 |  |
| T76 | Male | 58 | Follicular carcinoma | + | + | 8 | - | + |  |  |
| T77 | Female | 60 | PTC Follicular | + | - | 6 | - |  | Exon 19 deletion |  |
| T78 | Male | 59 | PTC columnar cell | + | + | 10 | - | + |  |  |
| T79 | Male | 55 | Follicular carcinoma | + | + | 7 | - |  | Exon 19 deletion |  |
| T80 | Female | 64 | PTC Classic | - | - | 4 | + |  |  |  |
| T81 | Female | 85 | PTC Classic | - | - | 5 |  |  |  | Low DNA |
| T82 | Female | 44 | PTC Follicular | + | - | 10 | - |  | 18 |  |
| T83 | Male | 66 | PTC Tall cell | + | + | 13 | - | + |  |  |
| T84 | Female | 60 | PTC Classic | - | - | 6 | + |  |  |  |
| T85 | Male | 57 | Follicular carcinoma | + | + | 7 | - |  |  |  |
| T86 | Female | 25 | PTC Classic | - | - | 3 | - |  | 21 |  |
| T87 | Female | 45 | PTC Classic | - | - | 3 | + |  |  |  |
| T88 | Male | 75 | PTC Follicular | + | + | 6 | + |  |  |  |
| T89 | Male | 45 | Follicular carcinoma | + | + | 8 | - |  | 21 |  |
| T91 | Male | 62 | Follicular carcinoma | + | + | 11 | - | + |  |  |
| T92 | Female | 85 | PTC Classic | + | - | 7 | + |  |  |  |
| T93 | Male | 80 | PTC Classic | + | - | 4 | - |  | Exon 19 deletion |  |
| T94 | Female | 35 | PTC Classic | - | - | 5 | + |  |  |  |
| T95 | Male | 63 | Follicular carcinoma | + | + | 13 | - | + |  |  |
| T96 | Female | 70 | PTC Classic | + | + | 3 | + |  |  |  |
| T97 | Female | 59 | PTC Classic | - | - | 4 |  |  |  | Necrosis |
| T98 | Male | 63 | PTC Follicular | + | + | 10 | - |  |  |  |
| T100 | Female | 48 | PTC Follicular | + | + | 6 | + |  |  |  |
| T102 | Male | 45 | PTC columnar cell | + | + | 8 | - |  | Exon 19 deletion |  |
| T103 | Male | 57 | PTC Classic | - | - | 3 |  |  |  | Low DNA |
| T104 | Male | 57 | PTC Tall cell | + | + | 11 | - | + |  |  |
| T105 | Female | 33 | PTC Follicular | - | - | 4 | - |  |  |  |
| T107 | Female | 30 | PTC Classic | - | - | 3 | + |  |  |  |
| T108 | Female | 43 | PTC Classic | + | + | 3 | + |  |  |  |
| TT1 | Male | 51 | Follicular carcinoma | + | + | 5 | - | + |  |  |
| TT2 | Male | 47 | Follicular carcinoma | + | + | 9 | - |  | 21 |  |
| TT3 | Female | 66 | PTC Classic | + | - | 2 | + |  |  |  |
| TT4 | Male | 69 | Follicular carcinoma | + | + | 8 | - |  |  |  |
| TT5 | Female | 44 | PTC Follicular | + | + | 5 | - |  |  |  |
| TT6 | Female | 52 | PTC Classic | + | + | 7 | + |  |  |  |
| TT7 | Female | 55 | PTC Follicular | + | + | 5 | - |  | Exon 19 deletion |  |
| TT8 | Male | 61 | Follicular carcinoma | + | + | 4 | - |  |  |  |
| TT9 | Male | 60 | PTC Follicular | + | + | 6 | - |  | Exon 19 deletion |  |
| TT10 | Female | 29 | PTC Classic | + | + | 3 | + |  |  |  |
| TT11 | Female | 39 | PTC Follicular | - | - | 2 | - | + |  |  |
| TT12 | Male | 62 | PTC columnar cell | + | + | 6 | - |  | 21 |  |
| TT13 | Female | 43 | PTC Classic | + | - | 4 | + |  |  |  |
| TT14 | Male | 48 | Follicular carcinoma | + | + | 10 | - |  | Exon 19 deletion |  |
| TT15 | Male | 41 | PTC Classic | - | - | 6 | + |  |  |  |
| TT17 | Female | 55 | PTC Follicular | + | + | 7 | - |  | 18 |  |
| TT18 | Male | 47 | Follicular carcinoma | + | + | 5 | - | + |  |  |
| TT19 | Female | 51 | PTC Classic | - | - | 3 | + |  |  |  |
| TT20 | Male | 77 | PTC Follicular | + | + | 5 | + |  |  |  |
| TT21 | Male | 48 | Follicular carcinoma | + | + | 8 | - |  | 21 |  |
| TT22 | Male | 56 | Follicular carcinoma | + | + | 11 | - | + |  |  |

*PTC= Papillary thyroid carcinoma

Table S2. *KRAS* and *EGFR* mutation status in *BRAF-V600E* negative samples

|  | *KRAS* mutation (+) | *KRAS* mutation (-) |
| --- | --- | --- |
| *EGFR* mutation (+) | 1 | 20 |
| *EGFR* mutation (-) | 18 | 7 |
